# Ruptured Gastrointestinal Stromal Tumor in a 12-Year-Old Child: A Case Report

**DOI:** 10.7759/cureus.93353

**Published:** 2025-09-27

**Authors:** Ashwin Rajkumar J, Prakash Agarwal, Madhu R, Jegadeesh Sundaram, Latha M Sneha

**Affiliations:** 1 Pediatric Surgery, Sri Ramachandra Institute of Higher Education and Research, Chennai, IND; 2 Pediatric Surgery, Apollo Hospitals, Chennai, IND; 3 Pediatric Hematology and Oncology, Sri Ramachandra Institute of Higher Education and Research, Chennai, IND

**Keywords:** cancer prognosis, gastrointestinal stromal tumor (gist), imatinib therapy, pediatric case, surgical emergencies

## Abstract

Gastrointestinal stromal tumors (GISTs) are rare mesenchymal neoplasms, especially in pediatric patients, often presenting with distinct clinical and molecular characteristics. This case report describes a 12-year-old girl with a large gastric GIST complicated by tumor rupture and hemoperitoneum. Initial treatment with imatinib was followed by emergent surgical intervention due to tumor bleeding. Histopathology confirmed an epithelioid-type GIST, necessitating continued imatinib therapy. Pediatric GISTs demonstrate an indolent but unpredictable course, requiring a multidisciplinary approach. Despite their aggressive nature, pediatric cases show better survival outcomes than adults. This case underscores the importance of timely intervention, individualized therapy, and long-term follow-up to improve prognosis in children with GISTs.

## Introduction

Gastrointestinal stromal tumors (GISTs) are rare, uncommon mesenchymal neoplasms arising from the interstitial cells of Cajal [1,2]. Pediatric GISTs differ significantly from adult cases in their clinical and molecular characteristics. While adult GISTs typically harbor receptor tyrosine kinase (KIT) or platelet-derived growth factor receptor alpha (PDGFRA) mutations and present with spindle cell histology, pediatric GISTs are often wild-type, more commonly show epithelioid morphology, and demonstrate variable response to tyrosine kinase inhibitors. Subtypes include KIT-mutant, PDGFRA-mutant, succinate dehydrogenase (SDH)-deficient, neurofibromatosis type 1 (NF1)-associated, and rare RAS pathway-driven variants, each with distinct biological and therapeutic implications [3]. Although most pediatric GISTs are sporadic, hereditary predispositions have been described, including familial cases with germline KIT or PDGFRA mutations, as well as associations with syndromes such as neurofibromatosis type 1 (NF1), Carney triad, and Carney-Stratakis syndrome. The primary distinction in pediatric GIST lies in increased resistance to chemotherapy and immunotherapy and the higher propensity for disseminated disease [4]. This warrants different handling and individualized therapy in children. We present a case of a 12-year-old girl presenting with a large GIST arising from the stomach with tumor rupture and hemoperitoneum.

## Case presentation

A 12-year-old girl, previously well, presented with complaints of abdominal pain and non-bilious vomiting for two weeks, with significant weight loss over the last six months. On examination, the child appeared malnourished and pale with tachycardia. Abdominal examination showed diffuse abdominal tenderness predominantly in the epigastric region with upper abdominal distension; no mass was palpable. Initial blood workup showed hemoglobin (Hb) of 9.6 g/dL with microcytic hypochromic anemia on peripheral smear (Table 1).

**Table 1:** Hematologic and biochemical investigations All values from the time of diagnosis to presentation and postoperative after surgical intervention. Reference ranges are based on institutional laboratory standards.

Parameters	Patient’s results at the time of diagnosis	Patient’s results at presentation	Patient’s results postoperatively	Reference range
Hemoglobin	9.6 g/dL	4.6 g/dL	9.1 g/dL	11-17 g/dL
Total leucocyte count	17,000 cells/mm^3^	6,500 cells/mm^3^	10,700 cells/mm^3^	4,000-17,000 cells/mm^3^
Platelet count	235,000 cells/mm^3^	136,000 cells/mm^3^	350,000 cells/mm^3^	150,000-450,000 cells/mm^3^
Blood urea nitrogen	12 mg/dL	10 mg/dL	12 mg/dL	6-20 mg/dL
Serum creatinine	0.4 mg/dL	0.6 mg/dL	0.5 mg/dL	0.7-1.2 mg/dL
Sodium	137 mEq/L	129 mEq/L	138 mEq/L	136-145 mEq/L
Potassium	4.2 mEq/L	3.6 mEq/L	4.5 mEq/L	3.5-5.1 mEq/L
Chloride	100 mEq/L	97 mEq/L	100 mEq/L	98-107 mEq/L
Bicarbonate	24 mEq/L	20 mEq/L	26 mEq/L	22-29 mEq/L

**Figure 1:**
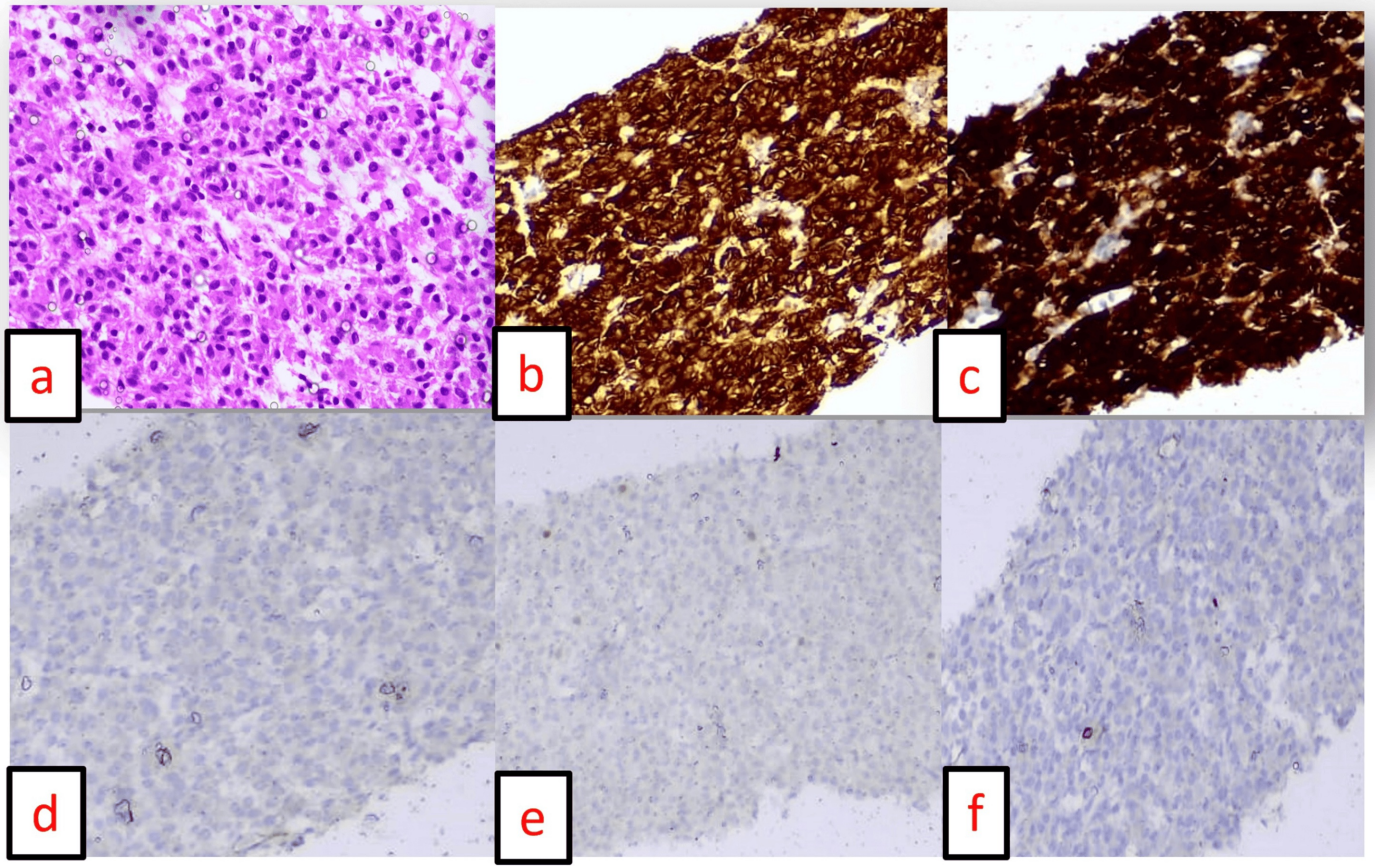
Immunohistochemistry of the core biopsy of the tumor a: Hematoxylin and eosin, ×400 (high power). b: DOG-1 positive, ×100. c: CD117 highly positive, ×100. d: PAN-CK negative, ×100. e: P63 negative, ×100. f: Desmin negative, ×100.

**Figure 2:**
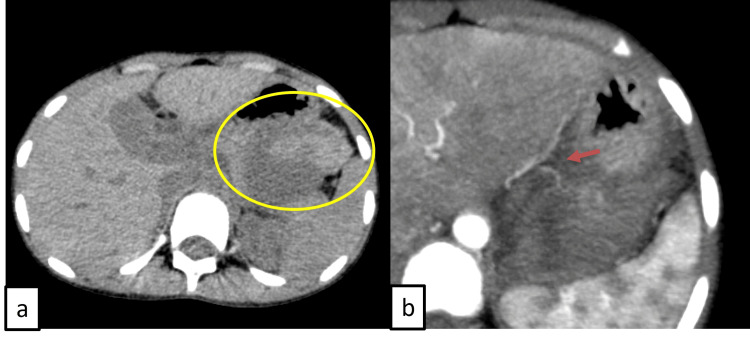
Preoperative computed tomography of the abdomen showing intralesional bleed a: Hyperdense area of hemorrhages (axial view). b: Left gastric artery feeder vessel.

Contrast-enhanced computed tomography (CT) of the abdomen revealed a well-defined heterogeneously enhancing soft tissue lesion measuring 9x5x4 cm arising from the posterior surface of the stomach with loss of fat plane, occupying the gastrosplenic space. The lesion also derived its arterial feeder from the branch of the left gastric artery and had no lymph node involvement. A CT-guided biopsy was done, which revealed linear cores of neoplastic tissue composed of monoclonal cells arranged in sheets with indistinct, moderately pale cytoplasm and uniformly round to oval nuclei. Immunohistochemistry (IHC) was strongly positive for CD117 and DOG-1, with a Ki67 of 8%, suggestive of gastrointestinal stromal tumor (Figure 1). Institutional tumor board discussion was done, and the patient was started on imatinib therapy. Although the diagnosis was established promptly once CT and biopsy with IHC were performed, the true diagnostic challenge in this case lay in the nonspecific initial symptoms (abdominal pain, vomiting, and weight loss without a palpable mass) and the extreme rarity of pediatric GIST, which can mimic more common conditions and delay clinical suspicion. The patient presented one week later with abdominal pain, vomiting, and hemodynamic instability. A significant drop in hemoglobin to 4.6 g/dL was noted, for which the child was adequately resuscitated (Table 1). Repeat CT of the abdomen (Figure 2) showed an interval increase in size of the lesion with intralesional hemorrhage and gross hemoperitoneum, following which she underwent emergency exploratory laparotomy.

**Figure 3:**
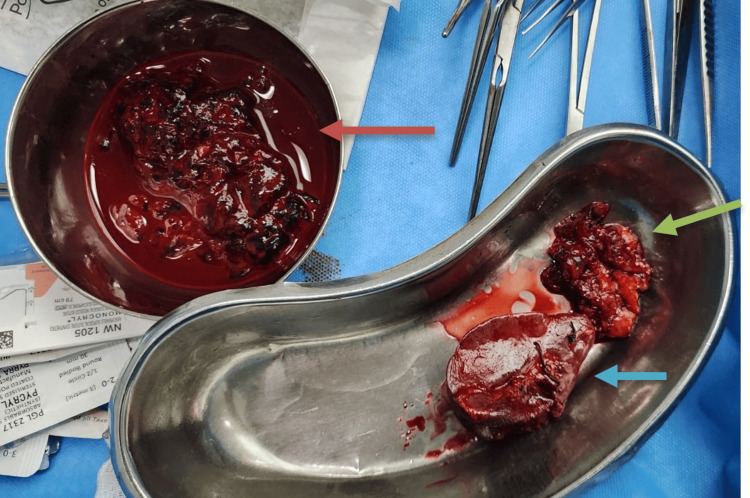
Intraoperative specimen The orange arrow depicts the piecemeal tumor. The blue arrow shows the resected spleen. The green arrow shows the resected specimen of the stomach.

Midline laparotomy revealed 1 L of gross hemorrhagic ascites with necrotic friable tumor within the lesser sac. The mass was adherent to the posterior wall of the stomach along the lesser curvature and superior aspect and tail of the pancreas, with erosion into the splenic pedicle with active bleeding. Intraoperative decision was taken to proceed with splenectomy to control the bleeding and wedge resection of the involved part of the stomach (Figure 3).

**Figure 4:**
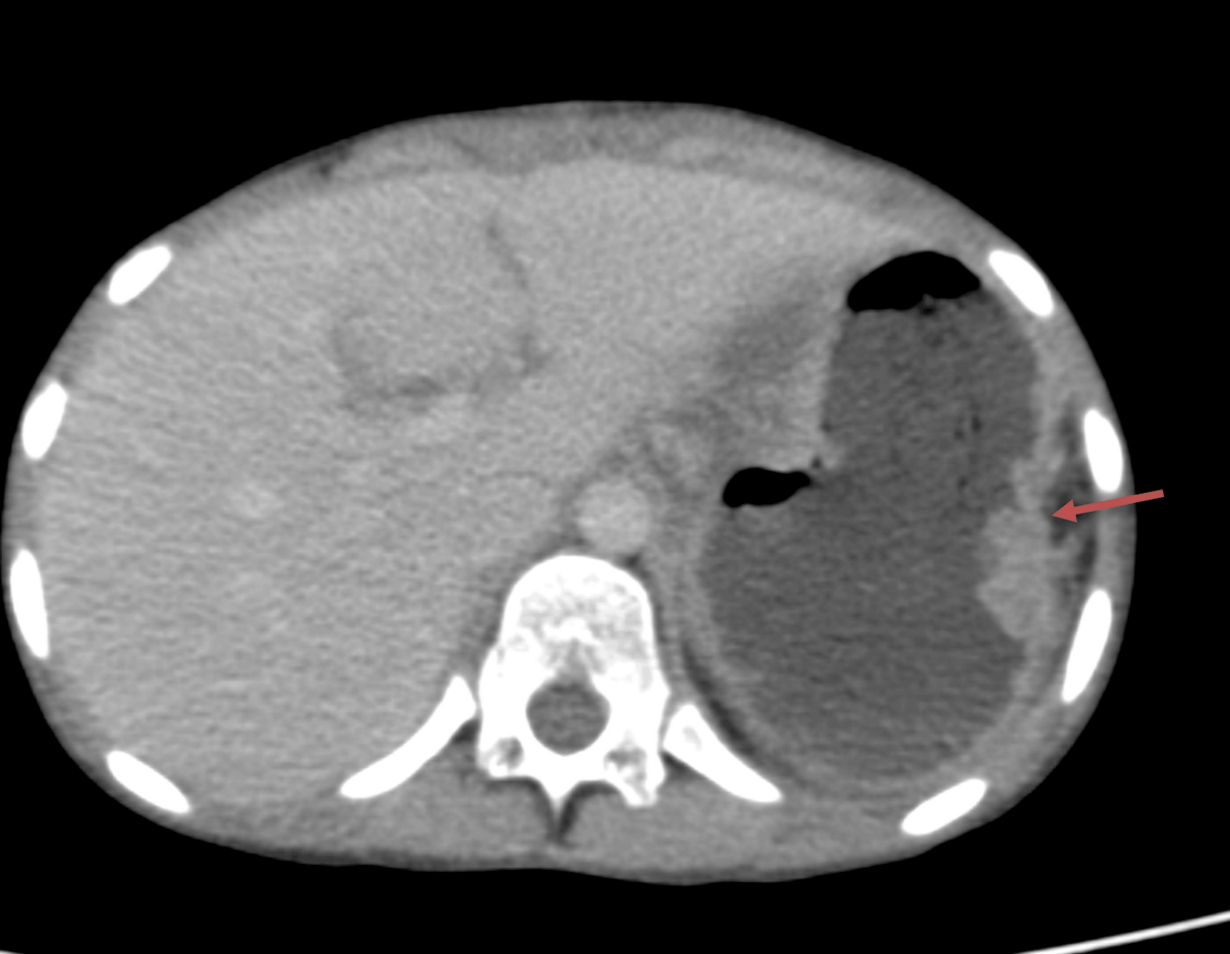
Postoperative computed tomography after six weeks showing mild thickening of the gastric wall

The postoperative period was uneventful and favorable in the ICU, with oral feeds started on day 5, and the patient was discharged on day 8. The resected specimen was an epithelioid type of gastrointestinal stromal tumor grade 1, with IHC strongly positive for CD117 and DOG-1, suggesting T3 disease, for which imatinib therapy was restarted after 14 days postoperatively. Postoperatively, after six weeks of treatment, a repeat CT of the abdomen was taken, showing mild thickening of the stomach wall (Figure 4).

## Discussion

The overall incidence of GISTs in children under the age of 14 is rare, with an incidence of 0.02 per million in the UK National Registry of Childhood Tumours [1,5]. Pediatric GISTs are more common in girls, with a median diagnosis age in the second decade of life, which either occur sporadically, as in our index case, or syndromically, associated with other tumor syndromes, such as neurofibromatosis type I, Carney triad, and Carney-Stratakis syndrome [5,6].

Sporadic GISTs are aggressive in children, often arising from the stomach with metastasis to the lymph nodes (29%), liver (25%), and peritoneum at diagnosis in 45% of cases [7-9]. Despite aggressive characteristics, these children show better survival outcomes than adults. The presenting signs and symptoms vary depending on the size and site of the lesion. These include abdominal pain, melena, hematemesis, and anemia caused by occult gastrointestinal bleeding [7,10,11]. Complications of GISTs are relatively uncommon, which include hemorrhage, obstruction, intussusception, or perforation, often necessitating surgical intervention [10,11]. Our case highlights one such rare presentation of tumoral rupture within the GISTs, warranting emergency surgery to alleviate symptoms. Notably, pediatric GISTs, despite their aggressive presentation, show better survival outcomes than adults, with reported five-year survival rates of 85%-90% in children compared to approximately 60%-70% in adults, underscoring the clinical significance of this case.

Complete surgical resection is standard practice for pediatric GIST, although frequent recurrence is seen despite curative resection and multiple surgeries [7,8,12,13]. Intraoperatively, a large friable tumor was identified, adherent to the posterior gastric wall along the lesser curvature and extending to the superior aspect of the pancreas. The lesion had invaded the splenic pedicle and was actively bleeding, necessitating emergency cytoreductive surgery in the form of tumor excision, wedge resection of the stomach, and splenectomy to achieve hemodynamic stability. In such cases, the aim is to alleviate symptoms to address immediate complications, rather than curative intent or long-term survival [7,8,12].

Spindle cells and epithelioid cells are the most common cell types in GIST, wherein epithelioid morphology predominates in pediatric GISTs [7,14]. Histopathology of the specimen revealed linear core neoplastic tissue with monoclonal cells strongly positive for CD117 and DOG-1 and negative for synaptophysin, desmin, and p63, suggestive of GIST, for which no standardized treatment protocol is described in the pediatric population as in adults [15,16]. Oral protein kinase inhibitor imatinib mesylate has been prescribed, which shows a response in 60% of cases despite having no c-Kit mutation and mutational variation, unlike the adult type [16]. Resistant cases are further followed up with other specific tyrosine kinase inhibitors such as sunitinib and regorafenib, providing alternative therapeutic strategies to the pediatric GIST population [7,16]. Although molecular analysis for c-kit, PDGFRA mutations, and NF1 status could not be performed in our patient due to logistic constraints, such genetic characterization is increasingly recognized as essential in guiding the choice of tyrosine kinase inhibitors and in tailoring future therapeutic strategies for pediatric GIST. In our case, on further tumor board discussion, it was decided to proceed with repeat imaging and surgical exploration, proceeding with either subtotal/ partial gastrectomy with Roux-en-Y gastrojejunostomy, depending on response to imatinib therapy for curative intent.

## Conclusions

Pediatric GISTs are rare and biologically distinct from adult cases, often requiring specialized management. This case highlights the challenges of diagnosis and the risk of rapid deterioration due to complications such as tumor rupture and hemorrhage, underscoring the necessity of a multidisciplinary approach involving pediatric oncologists, surgeons, and intensivists. Surgical intervention remains crucial for symptomatic relief and disease control, especially in cases of complications such as tumor rupture and hemoperitoneum. Targeted therapy with imatinib plays a key role in treatment, even in wild-type GISTs, although long-term outcomes remain uncertain. Regular follow-up with imaging and clinical assessment is essential to monitor disease progression and treatment response. This case underscores the importance of early recognition, timely intervention, and individualized treatment strategies to support better outcomes and quality of life in patients with pediatric GIST.
